# *FADS1-FADS2* gene cluster confers risk to polycystic ovary syndrome

**DOI:** 10.1038/srep21195

**Published:** 2016-02-16

**Authors:** Ye Tian, Wei Zhang, Shigang Zhao, Yinhua Sun, Yuehong Bian, Tailai Chen, Yanzhi Du, Jiangtao Zhang, Zhao Wang, Tao Huang, Yingqian Peng, Ping Yang, Han Zhao, Zi-Jiang Chen

**Affiliations:** 1 Center for Reproductive Medicine, Ren Ji Hospital, School of Medicine, Shanghai Jiao Tong University, Shanghai, China; 2 Shanghai Key Laboratory for Assisted Reproduction and Reproductive Genetics, Shanghai, China; 3 Center for Reproductive Medicine, Shandong Provincial Hospital Affiliated to Shandong University, Jinan, China; National Research Center for Assisted Reproductive Technology and Reproductive Genetics, China; The Key laboratory for Reproductive Endocrinology of Ministry of Education, China; Shandong Provincial Key Laboratory of Reproductive Medicine, Jinan, China; 4 Department of joint and bone oncology, Shandong Provincial Hospital Affiliated to Shandong University, Jinan, China

## Abstract

Dyslipidemia is common in polycystic ovary syndrome (PCOS). This study was aimed to investigate whether fatty acid desaturase genes (*FADS*), a dyslipidemia-related gene cluster, are associated with PCOS. We scanned variations of *FADS* genes using our previous data of genome-wide association study (GWAS) for PCOS and selected rs174570 for further study. The case-control study was conducted in an independent cohort of 1918 PCOS cases and 1889 age-matched controls and family-based study was conducted in a set of 243 core family trios with PCOS probands. Minor allele frequency (allele T) of rs174570 was significantly lower in PCOS cases than that in age-matched controls (*P* = 2.17E-03, OR = 0.85), even after adjustment of BMI and age. PCOS subjects carrying CC genotype had higher testosterone level and similar lipid/glucose level compared with those carrying TT or TC genotype. In trios, transmission disequilibrium test (TDT) analysis revealed risk allele C of rs174570 was significantly over-transmitted (*P* = 2.00E-04). Decreased expression of FADS2 was detected in PCOS cases and expression quantitative trait loci (eQTL) analysis revealed the risk allele C dosage was correlated with the decline of FADS2 expression (*P* = 0.002). Our results demonstrate that *FADS1-FADS2* are susceptibility genes for PCOS.

Polycystic ovary syndrome (PCOS) is the most common endocrine disorder affecting 5–10% women of reproductive age^1^. It is characterized by ovulatory dysfunction, clinical or biochemical hyperandrogenism and polycystic ovaries under ultrasound^2^. Affected women commonly present with higher risk of metabolic disorders, including dyslipidemia, obesity, insulin resistance and type 2 diabetes mellitus (T2DM)^3^,^4^,^5^. Dyslipidemia refers to a spectrum of abnormal lipid and lipoprotein profiles, such as elevated triglyceride (TG), low-density lipoprotein cholesterol (LDL-C) and total cholesterol (TC) concentrations and decreased high-density lipoprotein cholesterol (HDL-C) levels^6^. Lipid abnormalities are present in about 70% of non-Hispanic white women^6^ and 56.4% of Chinese women with PCOS^7^. A recent systematic review revealed that 28.6–95% of PCOS cases presented with decreased HDL-C level and 5.5–56% presented with increased triglycerides^4^. The presence of dyslipidemia implies an increase of cardiovascular risks and managements of lipid aberrations benefit long-term health of PCOS women^8^.

The etiology of PCOS is not very clear but studies have indicated that genetic factors are strongly involved^9^,^10^. Several candidate genes related with T2DM, insulin resistance and obesity have been proposed in order to identify novel PCOS susceptibility genes^11^,^12^,^13^,^14^. Results demonstrated that PCOS and these metabolic disorders might share some similar genetic predisposition in development. For example, T2DM-related gene *MTNR1B* and obesity-related gene *FTO* were found to be associated with PCOS^13^,^14^. Our PCOS GWAS also identified a novel PCOS candidate gene *THADA*, which was reported to be associated with T2DM^15^,^16^. However, few studies have evaluated dyslipidemia-related genes in PCOS cases.

*FADS1-FADS2-FADS3*, a cluster of fatty acid desaturase genes, are candidate genes of dyslipidemia which have been validated by a number of GWASs^17^,^18^,^19^,^20^,^21^,^22^. *FADS* genes encode key enzyme in metabolism of polyunsaturated fatty acids: fatty acid desaturase 1 (*FADS1*) encodes delta-5 (D5D), fatty acid desaturase 2 (*FADS2*) encodes delta-6 (D6D) desaturases^23^, and *FADS3* encodes a desaturase of unknown activity. This gene cluster is mapped to 11q12-q13.1, which was shown to be associated with concentration of TC, LDL-C, HDL-C and TG in numerous studies^17^,^18^,^19^,^20^,^21^,^22^. In above studies, common variant rs174570 is an important SNP marker. In 16 European population cohorts that included a total of 22562 individuals, rs174570 of *FADS2* gene was found to be associated with TC (*P* = 1.5E-10), LDL (*P* = 4.4E-13), HDL (*P* = 3.9E-06) and TG levels (*P* = 2.9E-05)^19^. Considering that dyslipidemia was common in PCOS cases, *FADS* genes may also confer risks to PCOS.

*FADS* genes are also associated with reproduction. The *FADS2* knockout female mouse is sterility and demonstrates impairment of follicle maturation and luteinization of stromal cells in ovaries^24^,^25^. Notably, *FADS* genes may also play a role in glucose metabolism. Studies have demonstrated that this gene cluster was associated with fasting glucose, homeostasis model assessment for insulin resistance, and insulin secretory capacity^26^,^27^. As insulin resistance and sterility are important features of PCOS, *FADS* genes may play a role in PCOS.

Altogether, considering overlapping genetic background between PCOS and dyslipidemia/diabetes and the potential gene function in reproduction, we sought to determine whether *FADS1-FADS2-FADS3*, validated dyslipidemia-related gene, are associated with PCOS or not. In the present work, after reviewing our previous discovery cohort of GWAS^16^, we investigated the association between rs174570 in *FADS* genes and PCOS.

## Results

### Basic clinical features

The clinical and metabolic characteristics of 1918 PCOS cases and 1889 age-matched controls were summarized in Table 1. The mean (±SD) age of PCOS cases and controls were 28.14 (±3.67) years and 28.32 (±3.67) years, respectively (*P* > 0.05). The mean body mass index (BMI) of PCOS cases was significantly higher than that of control group (24.94 ± 4.68 vs 22.49 ± 3.23, *P* < 0.001). Additionally, PCOS cases presented with higher testosterone (T) level than controls (*P* < 0.001).

In 243 family trios, the mean age of female offspring that affected with PCOS was 27.10 ± 3.86 years and the mean BMI was 25.04 ± 4.38 kg/m^2^. The mean T was 45.04 ± 36.84 ng/dl, mean FSH was 6.35 ± 1.54 IU/L, and mean LH was 11.10 ± 6.81 IU/L.

### SNP selection

**Figure 1:**
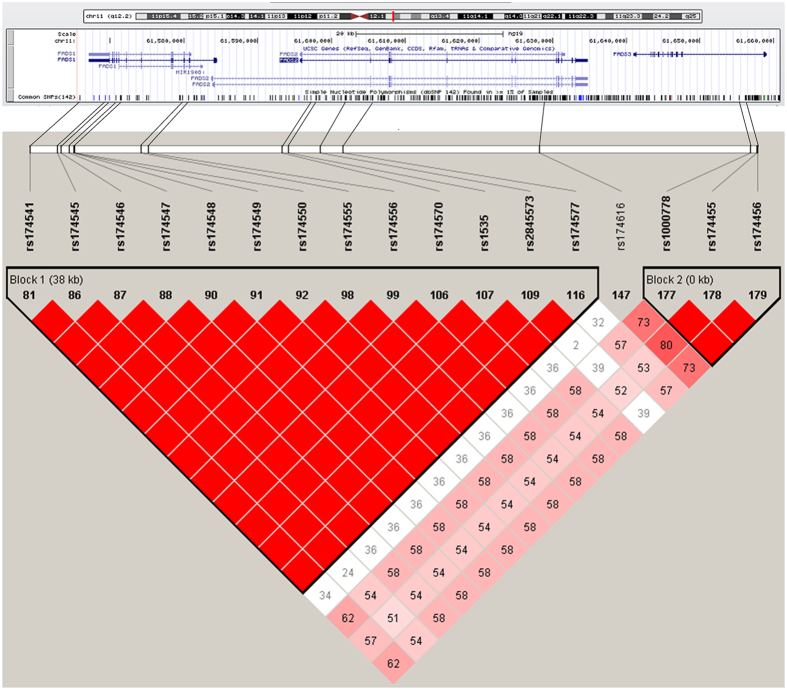
Linkage disequilibrium structure of 17 SNPs in *FADS* gene cluster in Chinese Han Beijing (CHB) population. Top portion of the Figure 1 shows the position of 17 SNPs (from UCSC Genome Browser http://genome.ucsc.edu/cgi-bin/hgGateway). Values in the box below show the LD value (D′) between the SNPs.

A total of 17 SNPs located in *FADS1-FADS2-FADS3* gene cluster were captured in our previous PCOS GWAS (See Supplemental Table S1). Linkage disequilibrium (LD) analysis was performed for these 17 SNPs in Chinese Han Beijing (CHB) population and two LD blocks were generated (See Fig. 1). SNPs in block 2 that cover *FADS3* were not significant in PCOS GWAS (P > 0.05). Most SNPs in block 1 that cover *FADS1-FADS2* gene were significant (P < 0.05). Rs174570, which is the most significant SNP in block 1, was selected to represent the whole block for subsequent study. And other two markers, rs174547 and rs1535 from block 1, were also genotyped to verify the association between *FADS1-FADS2* gene cluster and PCOS.

### Allele frequency comparison in case-control study

The genotype distribution of analyzed SNP was consistent with Hardy-Weinberg equilibrium (*P* > 0.05). Power reached 90% at α = 0.01, assuming risk allele frequency of 0.05 and genotype relative risk of 1.8. The allele frequency of rs174570 was summarized in Table 2. Minor allele frequency (MAF) of rs174570 was significantly lower in PCOS than that in control group (OR = 0.85, 95% CI 0.77–0.94, *P* = 2.17E-03). To eliminate the possible impact of age and BMI on the results, logistic regression was used for adjusting age and BMI and the difference was still significant (*P* = 4.47E-03). In order to obtain more solid results, we repeated the data by genotyping two other SNPs (rs1535, rs174547) in the same LD block with rs174570 and got similar results (see supplementary Table S2).

In order to eliminate the effect of lipid level on the association, the PCOS subjects were divided into normal lipid level groups and abnormal lipid level groups (see supplementary Table S3). PCOS subjects, whose TC, TG, HDL and LDL level were all normal, did not have significantly different MAF with PCOS subjects with abnormal lipid level (TC, TG, HDL or LDL level were abnormal). Genotype and allele frequencies of rs174570 did not differ significantly between two groups of PCOS cases divided by TC level (*P*_genotype_ = 0.963; *P*_allele_ = 0.785). The same trend occurred in different TG, LDL, and HDL groups (see supplemental Table S3).

### Endocrinal and metabolic measurements in PCOS of case-control study

Genotype comparison of rs174570 using three different genetic models was shown in supplemental Table S4. Genotype frequency differences of rs174570 were significant in additive model (*P* = 7.90E-03), in dominant model (*P* = 8.93E-03) and in recessive model (*P* = 1.66E-02). Additive model was selected for genotype-phenotype analysis in PCOS cases. PCOS subjects carrying CC genotype had a higher testosterone level compared with those carrying TT or TC genotype, although the difference was not significant (45.37 ± 28.21 vs 43.74 ± 19.23 vs 42.38 ± 17.81, *P* = 0.053). PCOS cases that carrying different copies of risk allele C of rs174570 exhibited no significant difference in FSH, LH, glucose or lipid-related traits, even adjusting for age and BMI (See Table 3).

### TDT analysis in PCOS trios

Genetic association in case-control study can be caused by population structure, environmental factors and genetic heterogeneity. One solution is the combination of family-based tests and case-control association analysis, which could provide a robust and powerful approach to identify susceptibility variants of PCOS.

Hardy-Weinberg equilibrium analysis found no deviation in trios study for rs174570 (*P* > 0.05). As shown in Table 2, significant difference in transmission was found for rs174570. This symmetrically distorted pattern of transmission strongly supports the association of *FADS* gene polymorphism with increased risk of PCOS. Moreover, the over-transmitted allele of rs174570 (allele C) was the same as the risk allele identified in the present case-control study and previous GWAS study.

To attenuate the impact of multiple testing, TDT result was adjusted by 10000 times permutation. The association between rs174570 and PCOS was still significant after correction for multiple testing (permutation *P* = 5.00E-04). TDT analysis was also performed for two additional SNPs (rs1535, rs174547) and the results were of same trend (see supplemental Table S2).

### eQTL analysis of rs174570

**Figure 2:**
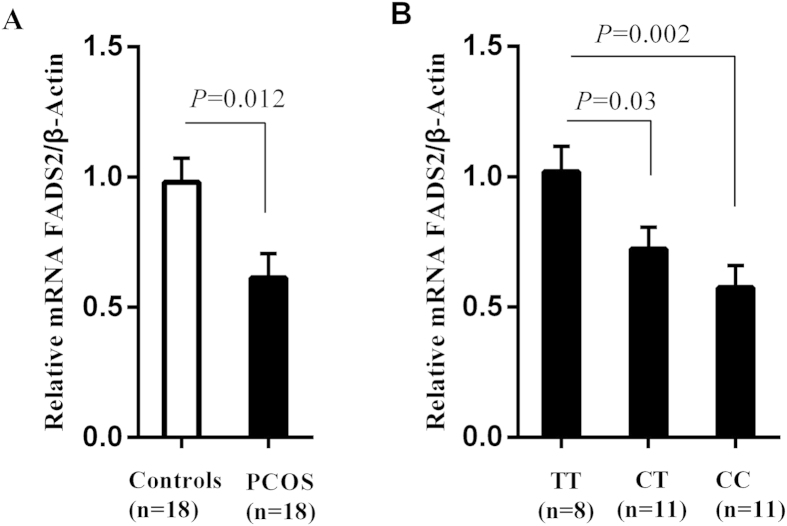
Genotype of rs174570 is associated with decreased mRNA expression of FADS2 in PCOS cases. The data are expressed as mean ± SE (standard error). (**A**) Quantitative real-time polymerase chain reaction (qRT-PCR) analysis of FADS2 mRNA levels in peripheral blood of 18 PCOS cases and 18 controls. The FADS2 mRNA level was normalized to β-actin and then adjusted by age and BMI using covariance analysis. (**B**) FADS2 mRNA levels in peripheral blood of 30 PCOS cases with TT, CT or CC genotypes, after normalization to β-actin and adjustment with age and BMI.

Rs17450 was located in *FADS2* gene. The expression of FADS2 in peripheral blood was decreased in PCOS cases compared with control group. After adjustment of age and BMI, the mean mRNA difference was still significant (*P* = 0.012, Fig. 2A). To evaluate potential effect of variant rs174570 on decreased FADS2 expression in PCOS, we compared mRNA levels of FADS2 among different genotypes (CC/CT/TT) in PCOS cases. FADS2 expression was associated with risk allele C dosage in liner regression models, with age and BMI included as a covariate (β = −0.218, *P* = 0.002). Samples with defined risk C allele revealed significantly decreased mRNA level of FADS2 (CT vs TT, *P* = 0.03; CC vs TT, *P* = 0.002. Fig. 2B), indicating that FADS2 expression in PCOS was associated with the copy number of allele C.

## Discussion

Based on the hypothesis that PCOS and metabolic traits share part of similar genetic background, testing candidate genes for metabolic traits in PCOS patients is a potential approach to identify PCOS susceptibility loci. This study focused on a dyslipidemia- and glucose metabolic-related *FADS1-FADS2* gene cluster, and showed for the first time that *FADS* gene confers risk to PCOS, independent of dyslipidemia and BMI. The results, obtained in a relatively large case-control study, were subsequently confirmed in a family-based study.

Common genetic variant rs174570 of *FADS1-FADS2* gene cluster is associated with PCOS, even after adjusting for age and BMI. Major allele C confers risks to PCOS because the allele frequency was higher in PCOS individuals of case-control association study and major allele C was over-transmitted in PCOS trios. We also compared the allele frequency of rs174570 within PCOS patients with different lipid profiles and indicated that this SNP is not associated with lipid in PCOS subjects. Considering the lack of lipid data from our control subjects, the possible spurious association via lipids could not be entirely excluded. Rs174570 is located in the intron region of *FADS2* and C allele is associated with decreased expression of FADS2 in PCOS cases. Hence, we speculate that rs174570 may regulate the expression of FADS2 directly or it is in strong LD with the true functional SNP. Considering rs174570 is located in the intron region, we then search for other functional variations in the same highly preserved LD block covering 48 kb genomic region^28^. Bokor *et al.* showed that minor allele of rs968567 that from the same LD block, was associated with higher FADS2 activity^29^. Moreover, rs968567 has been identified to be directly influence FADS2 expression. Rs968567 is located in the promoter region of *FADS2* and the presence of minor T allele increased promoter activity 2- to 3-fold compared with major C allele. Lattka *et al.* also identified that transcriptional factor ELK1 could bind to this site and the impact of ELK1 on FADS2 expression in carriers of minor T allele is higher^28^. It is possible that functional SNP among this LD block might explain the association between *FADS* genes and PCOS cases.

Compared with the wide type ovary which contains normal follicle development with multiple corpora lutea, ovary from *FADS2* knockout mouse lacks mature corpora lutea, indicating that *FADS2* may play a role in follicle maturation and ovulation, which are cardinal features of PCOS. In addition, inflammatory response may be another factor that links *FADS* variants with PCOS. Fatty acid metabolism may also play a role in the increased inflammatory state, which is thought to be an important factor for the development of PCOS. Fatty acid desaturases (FADS) can convert polyunsaturated fatty acids (PUFA) into precursor of inflammatory eicosanoids, including arachidonic acid (AA). Minor allele variants in *FADS1-FADS2* showed decreased production of arachidonic acid, which is precursor of pro-inflammatory eicosanoids such as prostaglandin E_2_ (PGE_2_)^30^,^31^,^32^,^33^. On the basis of these findings, PCOS cases with lower minor allele frequency are predicted to exhibit pro-inflammatory response, which is in accordance with chronic low-grade inflammation status in PCOS cases. It is possible that genetic variations in human *FADS* genes affect inflammation status and consequently act as PCOS risk factors. In this study, considering that PCOS affects the whole body and the difficulty of collecting tissue samples (such as ovaries or oocytes), we use peripheral blood to do the expression analysis of FADS2 in PCOS and controls. Given the existence of tissue specificity, eQTL analyses for PCOS subjects would ideally be conducted in ovaries. Hence, well-powered studies using ovaries or other related tissues are needed to repeat our results.

Of all examined glucose and lipid-related metabolic features in PCOS cases, rs174570 in *FADS1-FADS2* showed no association with fasting glucose, fasting insulin, homeostasis model for insulin resistance (HOMA-IR), cholesterol, triglycerides, high density lipoprotein and low density lipoprotein, even after BMI adjustment. Firstly, selected PCOS cases are of reproductive age and lipid levels can vary considerably with age^34^,^35^. In PCOS cases, the rate of hyperlipidemia increases with age^7^. Secondly, it is also hypothesized that this may result from different dietary intake of people in different regions. For example, the prevalence of metabolic syndrome was reported to be lower in Chinese women with PCOS than that in US women^7^,^36^ and the dietary structure difference was considered as an important factor. Moreover, we also speculate that the effects of *FADS* variants on PCOS and lipid/glucose metabolism are mediated by different mechanisms. PCOS subjects carrying CC genotype had higher testosterone level compared with those carrying TT or TC genotype, although the difference was not significant. There’s no published literature assessing the relation between FADS and testosterone level. Accumulated evidence suggests that FADS2 plays a crucial role in insulin resistance^37^,^38^,^39^, which is related with testosterone in PCOS. However, in this study, variation in *FADS2* showed no association with HOMA-IR. Further studies are needed to elucidate the role of *FADS* genes in testosterone level.

This study presented opportunities for future exploration of other lipid metabolic-related genes in PCOS cases. However, genetic loci that contributed to metabolic traits demonstrated mixed results in associations with PCOS. A recently candidate-wide association study (CWAS) detected SNPs in susceptibility loci of several traits such as lipid levels, type 2 diabetes and obesity in PCOS cases. None of the metabolic-related SNPs was associated with PCOS after strict correction for multiple testing^40^. The mixed results on association between lipid metabolic-related genes and PCOS might be explained on several aspects: different races of subjects, different sample sizes and different control groups (community controls/strict controls). In this study, the utilized sample size is relatively large, including 744 PCOS and 895 controls from previous GWAS study^16^, 1918 PCOS and 1889 controls for replication, and 243 trios, which were all pure Han Chinese. Additionally, all the controls in this study are strict controls rather than community controls. In the future, well-designed studies with large sample size are warranted to confirm our findings and studies to exam other validated dyslipidemia-related genes in PCOS cases are needed.

In conclusion, we identified *FADS* genes, which involved in lipid/glucose metabolism and inflammatory status, is a novel candidate gene cluster for PCOS.

## Method

### Subjects

PCOS diagnosis was according to the 2003 Rotterdam PCOS consensus criteria^2^ with at least two of the following features: oligo-ovulation or aovulation, clinical or biochemical hyperandrogenism (Ferriman-Gallwey score ≥6 or elevated circulating total testosterone ≥60 ng/dl)^16^,^41^, and polycystic ovaries morphology on ultrasound. Other related diseases, such as congenital adrenal hyperplasia, androgen-secreting tumors, Cushing’s syndrome, thyroid disease and hyperprolactinaemia, were excluded. The inclusion criteria for the control group were as follows: normal menstrual cycles, neither hyperandrogenism nor polycystic ovaries (PCO) under ultrasound. All individuals who were taking medications such as oral contraceptives and metformin during last 3 months were excluded.

A total of 1918 PCOS cases and 1889 age-matched unrelated controls were recruited consecutively from 2011 to 2013 at the Center for Reproductive Medicine, Provincial Hospital Affiliated to Shandong University and the Center for Reproductive Medicine, Renji Hospital, School of Medicine, Shanghai Jiaotong University. PCOS cases and controls were independent of subjects in our previous GWAS^16^.

A set of 243 Han Chinese core family trios, consisting of mothers, fathers and offspring affected with PCOS, were enrolled at the same centers. None of the PCOS probands originated from previous GWAS or 1918 replication cases. Written informed consents were obtained from all participates and this study was approved by Institutional Review Board of Shandong University and Shanghai Jiaotong University. All methods were carried out in accordance with the approved guidelines.

### Biochemical measurements

Serum testosterone (T) level of subject was measured during days 2–4 of the menstrual cycle by a chemiluminescent analyser (Beckman Access Health Company, Chaska, Minnesota, USA). Fasting glucose was measured by AU640 automatic biochemistry analyser (Olympus Company, Hamburg, Germany) and insulin was measured by chemiluminescent analyzer. Insulin resistance was calculated as fasting glucose (mmol/L) × fasting insulin (mIU/L)/22.5 using homeostasis model assessment (HOMA-IR). Serum TC, TG, LDL-C and HDL-C were detected by Ft-7060 (Beckman Coulter Inc, Galway, Ireland).

Continuous variables of clinical characteristics of PCOS cases and controls were presented as mean ± SD. According to the National Cholesterol Education Program criteria^42^ and Chinese guidelines on prevention and treatment of dyslipidemia in adults^43^: normal TC level < 5.18mmol/L, abnormal TC level ≥ 5.18 mmol/L; normal TG level < 1.7mmol/L, abnormal TG level ≥ 1.7 mmol/L; normal LDL-C level < 3.37mmol/L, abnormal LDL-C level ≥ 3.37 mmol/L; normal HDL-C level ≥ 1.04mmol/L, low HDL-C level < 1.04 mmol/L.

### SNP genotyping

Genomic DNA was extracted from whole peripheral blood using QIAamp DNA mini kit (Qiagen, Hilden, Germany). Genotyping of SNPs was carried out by sequenom assay (Bioyong Technologies Inc. Beijing, China) or directly sequencing.

### Analysis of genetic association data

The genetic power of case-control and family-based studies were analyzed by Genetic Power Calculator (http://pngu.mgh.harvard.edu/purcell/gpc). PLINK v.1.07 (http://pngu.mgh.harvard.edu/purcell/plink) was applied for the Hardy-Weinberg equilibrium calculation. In case-control study, allele frequency comparisons were performed by PLINK v.1.07 and then adjusted by BMI and age with logistic regression.

Genetic models were divided into additive (+/+ vs. +/− vs. −/−), dominant (+/+ plus +/− vs. −/−) and recessive (+/+ vs. +/− plus −/−). In genotype-phenotype analysis for PCOS cases, additive model was selected and liner regression analysis was used with SPSS v.22.0 software (SPSS Inc., Chicago, IL, USA).

### TDT analysis

In family-based study, the transmission disequilibrium test (TDT) was used to detect the association between SNP and PCOS with Haploview 4.2 software. Details of TDT were described in our previous study^44^. The TDT is a valid chi-square test in which unrelated family trios with PCOS were recruited and the probability of parents-to-offspring transmission was analyzed. If the probability of transmission was 50%, no association was assumed. On the contrary, if a discrepancy exists, the reason would be an association between gene polymorphisms and PCOS. Permutation function of Haploview was used for further correction of multiple testing.

### eQTL analysis

Total mRNA was isolated from whole peripheral blood using RNAiso Plus (Takara). cDNA was synthesized from total RNA using PrimeScript^TM^ RT reagent Kit with gDNA Eraser (Perfect Real Time, Takara). The expression levels of FADS2 mRNA were examined by Roche Lightcycle 480 using SYBR Green I Master (Roche). The relative FADS2 mRNA level was normalized to β-Actin using the comparative cycles at threshold fluorescence (Ct) method. The primer sequences of FADS2 and β-Actin were listed in Supplementary Table S5. The eQTL analysis was performed based on risk allele dosage and fitted linear regression models, with age and BMI included as a covariate. FADS2 mRNA mean levels comparison between PCOS cases and controls (or between any two genotype groups of PCOS) were analyzed by covariance analysis using SPSS v.22.0 software, and age and BMI were adjusted. P < 0.05 was regarded as statistically different.

## Additional Information

**How to cite this article**: Tian, Y. *et al.* *FADS1-FADS2* gene cluster confers risk to polycystic ovary syndrome. *Sci. Rep.* **6**, 21195; doi: 10.1038/srep21195 (2016).

## Supplementary Material

Supplementary Information

## Figures and Tables

**Table 1:** Characteristics of PCOS cases and controls.

	Cases	Controls	*P*
Age (years)	28.14 ± 3.67	28.32 ± 3.67	0.131
BMI (kg/m^2^)	24.94 ± 4.68	22.49 ± 3.23	<0.001
FSH (IU/L)	6.23 ± 1.72	6.96 ± 2.33	<0.001
LH (IU/L)	10.49 ± 6.12	5.05 ± 2.40	<0.001
T (ng/dl)	44.02 ± 23.81	28.31 ± 13.78	<0.001

BMI: body mass index. FSH: follicle stimulating hormone. LH: luteinizing hormone. T: testosterone.

**Table 2:** Allele frequency comparison and TDT analysis of rs174570.

SNP	Gene	Case-control study				Trios study				
						MAF		*P*	OR (95% CI)	Over-T	T/ Not-T	T-freq	TDT *χ*2	*P*
						PCOS	Control							
rs174570 T/C^a^	*FADS2*	0.273	0.306	2.17E-03	0.86 (0.77–0.94)	C	122/71	0.632	13.477	2.00E-04

^a^Minor allele/major allele. MAF: minor allele frequency. ORs: Odds Ratios. 95% CI: confidence interval. OR is for the minor allele. Over-T: over-transmitted allele. T: number of transmissions in TDT analysis. Not-T: number of un-transmissions in TDT analysis. T-freq: over-transmitted allele frequency.

**Table 3:** Endocrinal and metabolic characteristics comparison of rs174570 T/C using additive model in PCOS cases.

	TT(n = 132)	TC(n = 745)	CC(n = 968)	BETA	P	BETA-adj	P-adj
FSH (IU/L)	6.35 ± 1.88	6.15 ± 1.89	6.26 ± 1.56	0.028	0.681	0.035	0.611
LH (IU/L)	10.87 ± 5.92	10.43 ± 6.46	10.41 ± 5.69	−0.129	0.589	−0.125	0.592
T (ng/dl)	43.74 ± 19.23	42.38 ± 17.81	45.37 ± 28.21	1.843	0.053	1.629	0.085
F-GLU (mmol/l)	5.57 ± 1.26	5.48 ± 0.88	5.48 ± 0.91	−0.023	0.535	−0.021	0.554
F-INS (mIU/L)	13.78 ± 7.90	13.72 ± 8.70	13.96 ± 9.29	0.163	0.649	0.054	0.845
HOMA-IR	3.58 ± 2.85	3.45 ± 2.62	3.50 ± 2.74	0.003	0.977	0.048	0.590
TC (mmol/l)	4.46 ± 0.85	4.51 ± 0.87	4.52 ± 0.82	0.019	0.577	0.020	0.524
TG (mmol/l)	1.35 ± 0.70	1.42 ± 1.37	1.38 ± 1.25	−0.014	0.789	−0.019	0.704
HDL-C (mmol/l)	1.31 ± 0.29	1.33 ± 0.32	1.31 ± 0.30	−0.011	0.354	−0.008	0.501
LDL-C (mmol/l)	3.18 ± 0.97	3.11 ± 0.90	3.12 ± 0.90	−0.013	0.718	−0.018	0.591

BETA: the effect on the clinical and metabolic traits for each copy of the risk allele (allele C). BETA-adj: the effect on the clinical and metabolic traits for each copy of the risk allele after age and BMI adjustment. P-adj: adjusted P value by age and BMI using logistic regression. FSH: follicle stimulating hormone. LH: luteinizing hormone. T: testosterone.F-GLU: fasting glucose. F-INS: fasting insulin. HOMA-IR: homeostasis model for insulin resistance. CHOL: cholesterol. TG: triglycerides. HDL-C: high density lipoprotein. LDL-C: low density lipoprotein.
