# Supplementary material for: FADS1-FADS2 gene cluster confers risk to polycystic ovary syndrome
Source: Sci Rep. 2016 Feb 16;6:21195. doi: 10.1038/srep21195 (PMC4754766; doi:10.1038/srep21195)
Supplement: Supplementary Information [file srep21195-s1.doc]

**Title:** *FADS1-FADS2* gene cluster confers risk to polycystic ovary syndrome

**Authors’ names and institutions**:

**Ye Tian^1, 2, 3^, Wei Zhang^4^, Shigang Zhao^1,2^, Yinhua Sun^3^, Yuehong Bian^3^, Tailai Chen^3^, Yanzhi Du^1,2^, Jiangtao Zhang^3^, Zhao Wang^3^, Tao Huang^3^, Yingqian Peng^3^, Ping Yang^3^, Han Zhao^3*^, Zi-Jiang Chen^1,2,3^**

^1^Shanghai Key Laboratory for Assisted Reproduction and Reproductive Genetics,

**^2^**Center for Reproductive Medicine, Renji Hospital, School of Medicine, Shanghai Jiao Tong University, Shanghai, China

**^3^**Center for Reproductive Medicine, Provincial Hospital Affiliated to Shandong University, Jinan, China; National Research Center for Assisted Reproductive Technology and Reproductive Genetics, China; The Key laboratory for Reproductive Endocrinology of Ministry of Education, China; Shandong Provincial Key Laboratory of Reproductive Medicine, Jinan, China.

**^4^**Department of joint and bone oncology, Provincial Hospital Affiliated to Shandong University, Jinan, China.

**Supplemental table S1: Allele frequencies comparison of 17 SNPs in *FADS* gene cluster in previous PCOS GWAS.**

|  |  |  |  | MAF | |  |  |
| --- | --- | --- | --- | --- | --- | --- | --- |
| SNP | Gene | Allele ^a^ | Position | PCOS | Control | *P* | OR |
| rs174541 | *FEN1/FADS1* | C/T | 61798436 | 0.2596 | 0.2964 | 1.81E-02 | 0.8323 |
| rs174545 | *FADS1* | G/C | 61801834 | 0.2586 | 0.2961 | 1.60E-02 | 0.8292 |
| rs174546 | *FADS1* | T/C | 61802358 | 0.2586 | 0.2961 | 1.60E-02 | 0.8292 |
| **rs174547** | ***FADS1*** | **C/T** | **61803311** | **0.2613** | **0.2993** | **1.50E-02** | **0.8279** |
| rs174548 | *FADS1* | G/C | 61803876 | 0.2553 | 0.2907 | 2.22E-02 | 0.8364 |
| rs174549 | *FADS1* | A/G | 61803910 | 0.256 | 0.2907 | 2.48E-02 | 0.8393 |
| rs174550 | *FADS1* | C/T | 61804006 | 0.2583 | 0.2961 | 1.51E-02 | 0.8278 |
| rs174555 | *FADS1* | C/T | 61812288 | 0.255 | 0.2903 | 2.27E-02 | 0.8368 |
| rs174556 | *FADS1* | T/C | 61813163 | 0.255 | 0.2893 | 2.68E-02 | 0.8409 |
| **rs174570** | ***FADS2*** | **T/C** | **61829740** | **0.241** | **0.2933** | **8.18E-04** | **0.7652** |
| **rs1535** | ***FADS2*** | **G/A** | **61830500** | **0.2557** | **0.2955** | **1.07E-02** | **0.8192** |
| rs2845573 | *FADS2* | T/A | 61834436 | 0.1862 | 0.224 | 7.14E-03 | 0.7924 |
| rs174577 | *FADS2* | A/C | 61837342 | 0.2613 | 0.297 | 2.22E-02 | 0.8374 |
| rs174616 | *FADS2* | A/G | 61861650 | 0.1513 | 0.158 | 5.97E-01 | 0.9504 |
| rs1000778 | *FADS3* | A/G | 61887833 | 0.1871 | 0.188 | 9.47E-01 | 0.9941 |
| rs174455 | *FADS3* | G/A | 61888645 | 0.2374 | 0.2546 | 2.51E-01 | 0.9115 |
| rs174456 | *FADS3* | C/A | 61888710 | 0.1939 | 0.1894 | 7.44E-01 | 1.029 |

a: Minor allele/major allele. MAF: minor allele frequency. ORs: Odds Ratios. OR is for the minor allele.

**Supplemental table S2: Allele frequency comparison and TDT analysis of rs1535 and rs174547**

|  | Gene | Case-control study | | | | Trios study | | | | |
| --- | --- | --- | --- | --- | --- | --- | --- | --- | --- | --- |
| SNP |  | MAF | | *P* | OR  (95% CI) | Over-T | T/ Not-T | T-freq | TDT χ^2^ | *P* |
|  |  | PCOS | control |  |  |  |  |  |  |  |
| rs1535 G/A^a^ | *FADS2* | 0.282 | 0.314 | 2.66E-03 | 0.8592  (0.78-0.95) | A | 126/71 | 0.640 | 15.355 | 8.91E-05 |
| rs174547 C/T | *FADS1* | 0.286 | 0.314 | 8.15E-03 | 0.8753  (0.79-0.97) | C | 125/71 | 0.638 | 14.878 | 1.00E-04 |

| a: Minor allele/major allele. MAF: minor allele frequency. ORs: Odds Ratios. 95% CI: confidence interval. OR is for the minor allele. Over-T: over-transmitted allele. T: number of transmissions in TDT analysis. Not-T: number of un-transmissions in TDT analysis. T-freq: over-transmitted allele frequency. |
| --- |

**Supplemental table S3: Genotype and allele** frequencies comparison of rs174570 in PCOS women divided to normal lipid group and abnormal lipid group.

| Lipid group | CC | CT | TT | C | T | *P*-genotype | *P*-allele |
| --- | --- | --- | --- | --- | --- | --- | --- |
| TC,TG,LDL-C and HDL-C all normal | 410 | 332 | 57 | 1152 | 446 |  |  |
| TC or TG or LDL-C or HDL-C abnormal | 448 | 318 | 56 | 1214 | 430 | 0.434 | 0.261 |
| TC <5.18mmol/L(normal) | 692 | 520 | 90 | 1904 | 700 |  |  |
| TC ≥5.18mmol/L(abnormal) | 168 | 130 | 23 | 466 | 176 | 0.963 | 0.785 |
| TG <1.7mmol/L(normal) | 664 | 516 | 84 | 1844 | 684 |  |  |
| TG ≥1.7mmol/L(abnormal) | 196 | 134 | 29 | 526 | 192 | 0.386 | 0.866 |
| LDL-C < 3.37mmol/L(normal) | 548 | 422 | 72 | 1518 | 566 |  |  |
| LDL-C ≥ 3.37mmol/L(abnormal) | 312 | 228 | 41 | 852 | 310 | 0.885 | 0.767 |
| HDL-C ≥ 1.04 mmol/L(normal) | 745 | 582 | 98 | 2072 | 778 |  |  |
| HDL-C < 1.04 mmol/L(abnormal) | 115 | 68 | 15 | 298 | 98 | 0.217 | 0.284 |

TC: total cholesterol. TG: triglycerides. LDL: low density lipoprotein. HDL: high density lipoprotein

| **Supplemental table S4: Genotype frequency comparison of rs174570 in PCOS and controls using different genetic models.**   | SNP | Comparisons | PCOS | Control | P | | --- | --- | --- | --- | --- | | rs174570 T/C | ADD | 126/715/917 | 170/758/857 | 7.90E-03 | |  | DOM | 841/917 | 928/857 | 8.93E-03 | |  | REC | 126/1632 | 170/1615 | 1.66E-02 |   SNP, presented as minor allele/major allele. ADD, additive genotype model (+/+ vs. +/- vs. -/-). DOM, dominant genotype model (+/+ plus+/- vs. -/-). REC, recessive genotype model (+/+ vs. +/- plus -/-). |
| --- | --- | --- | --- | --- | --- | --- | --- | --- | --- | --- | --- | --- | --- | --- | --- | --- | --- | --- | --- | --- |

**Supplemental table S5:** Primer sequences of FADS2 and β-Actin

| Genes | Primers |
| --- | --- |
| *FADS2* | F- AAGGGTGCCTCTGCCAACT |
|  | R- GATTGTAGGGCAGGTATTTCAGC |
| *β-Actin* | CGACAGGATGCAGAAGGAG |
|  | ACATCTGCTGGAAGGTGGA |

F: forward. R: reward.
